# {2,2′-[(2,2-Dimethyl­propane-1,3-di­yl)bis­(nitrilo­methyl­idyne)]diphenolato}palladium(II) ethanol hemisolvate

**DOI:** 10.1107/S1600536808017078

**Published:** 2008-06-13

**Authors:** Wan Nazihah Wan Ibrahim, Mustaffa Shamsuddin, Suchada Chantrapromma, Hoong-Kun Fun

**Affiliations:** a Department of Chemistry, Faculty of Science, Universiti Teknologi Malaysia, 81310 UTM Skudai, Johor, Malaysia; b Department of Chemistry, Faculty of Science, Prince of Songkla University, Hat-Yai, Songkhla 90112, Thailand; c X-ray Crystallography Unit, School of Physics, Universiti Sains Malaysia, 11800 USM, Penang, Malaysia

## Abstract

The asymmetric unit of the title complex, [Pd(C_19_H_20_N_2_O_2_)]·0.5C_2_H_5_OH, contains two mol­ecules of a Pd^II^ complex of a Schiff base ligand with an N_2_O_2_ donor set and one ethanol mol­ecule. The Pd^II^ centers are in distorted square-planar geometries with the N_2_O_2_ donor atoms of the tetra­dentate Schiff base dianions. The ethanol mol­ecule takes part in an O—H⋯O hydrogen bond. In the crystal structure, mol­ecules are stacked approximately along the *b*-axis direction. The O atom and three H atoms of the solvent molecule are disordered over two positions; the site occupancy factors are *ca* 0.8 and 0.2.

## Related literature

For related structures, see, for example: Adrian *et al.* (2008 ▶). For background to applications of palladium(II) complexes, see, for example: Abu-Surrah *et al.* (1999 ▶); Adrian *et al.* (2008 ▶); Ayala *et al.* (2004 ▶); Caselli *et al.* (2005 ▶); Lai *et al.* (2005 ▶); Pou *et al.* (2007 ▶); Ramírez *et al.* (2008 ▶); Roy *et al.* (2008 ▶). For bond-length data, see: Allen *et al.* (1987 ▶). For ring puckering parameters, see: Cremer & Pople (1975 ▶).
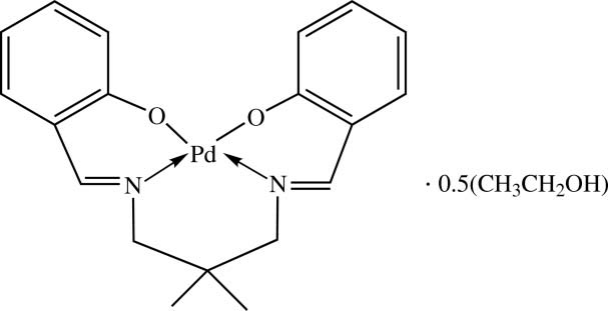


## Experimental

### 

#### Crystal data

[Pd(C_19_H_20_N_2_O_2_)]·0.5C_2_H_6_O
*M* *_r_* = 437.83
Monoclinic, 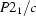

*a* = 12.2453 (3) Å
*b* = 13.7334 (3) Å
*c* = 22.8442 (5) Å
β = 101.092 (1)°
*V* = 3769.94 (15) Å^3^
*Z* = 8
Mo *K*α radiation
μ = 1.00 mm^−1^
*T* = 296 (2) K
0.38 × 0.33 × 0.23 mm

#### Data collection

Bruker SMART APEXII CCD area-detector diffractometer
Absorption correction: multi-scan (*SADABS*; Bruker, 2005 ▶) *T*_min_ = 0.690, *T*_max_ = 0.804
93296 measured reflections
11002 independent reflections
8985 reflections with *I* > 2σ(*I*)
*R*_int_ = 0.039

#### Refinement

*R*[*F* ^2^ > 2σ(*F* ^2^)] = 0.042
*wR*(*F* ^2^) = 0.130
*S* = 1.13
11002 reflections
474 parameters
H-atom parameters constrained
Δρ_max_ = 0.81 e Å^−3^
Δρ_min_ = −0.66 e Å^−3^

### 

Data collection: *APEX2* (Bruker, 2005 ▶); cell refinement: *APEX2*; data reduction: *SAINT* (Bruker, 2005 ▶); program(s) used to solve structure: *SHELXTL* (Sheldrick, 2008 ▶); program(s) used to refine structure: *SHELXTL*; molecular graphics: *SHELXTL*; software used to prepare material for publication: *SHELXTL* and *PLATON* (Spek, 2003 ▶).

## Supplementary Material

Crystal structure: contains datablocks global, I. DOI: 10.1107/S1600536808017078/ez2126sup1.cif

Structure factors: contains datablocks I. DOI: 10.1107/S1600536808017078/ez2126Isup2.hkl

Additional supplementary materials:  crystallographic information; 3D view; checkCIF report

## Figures and Tables

**Table 1:** Hydrogen-bond geometry (Å, °)

*D*—H⋯*A*	*D*—H	H⋯*A*	*D*⋯*A*	*D*—H⋯*A*
O5*A*—H5*AB*⋯O1*A*^i^	0.82	2.33	3.020 (13)	142

Symmetry code: (i) 
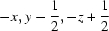
.

**Fig. 1.**
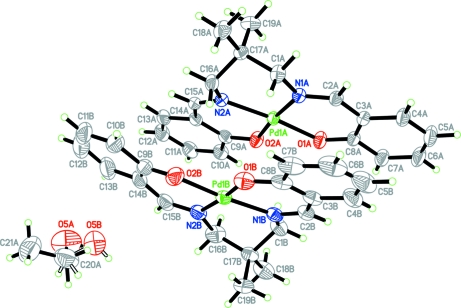
The asymmetric unit of (I), showing 50% probability displacement ellipsoids and the atomic numbering. Disorder of the ethanol molecule is shown.

**Fig. 2.**
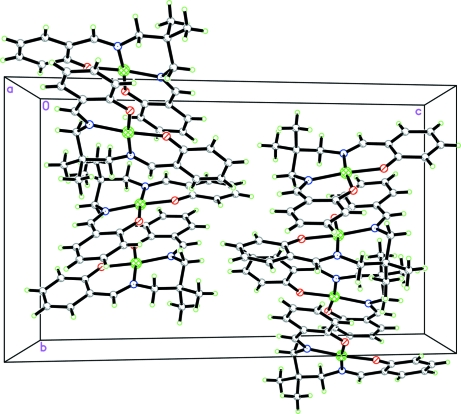
Crystal packing of (I), viewed along the a axis showing the stacking of the molecules along the b direction. The disordered ethanol molecule is omitted for clarity.

## supplementary crystallographic information

### Comment

In the field of coordination chemistry, the unique properties of Schiff bases as chelating ligands have attracted significant attention (Caselli *et al.*, 2005; Pou *et al.*, 2007; Ramírez *et al.*, 2008; Roy *et al.*, 2008). Their complexes with palladium(II) ions are found to be efficient catalysts in organic synthesis, especially in C—C bond formation (Abu-Surrah *et al.*, 1999; Ayala *et al.*, 2004; Lai *et al.*, 2005). In the present paper, the preparation and crystal structure of the complex *N*,*N*'-bis-salicylidene-2,2-dimethylpropane-1,3-diamine palladium(II) is described.

The asymmetric unit of the title complex (Fig. 1) contains two molecules of the Pd^II^ complex (*A* and *B*) of the Schiff base ligand and one solvated ethanol molecule. The ethanol molecule shows disorder. The Pd^II^ ion in both *A* and *B* has a distorted square-planar environment in which the ligand is coordinated to the Pd^II^ ion as a tetradentate chelating ligand *via* the two phenolic oxygen atoms and two imine nitrogen atoms, yielding three six-membered rings. In *A*, two rings are essentially planar (Pd1A/N1A/C2A/C3A/C8A/O1A and Pd1A/N2A/C9A/C14A/C15A/O2A) and one adopts a half-chair conformation; Pd1A/N1A/C1A/C17A/C16A/N2A with atom C17A displaced from the Pd1A/N1A/C1A/C16A/N2A plane by 0.425 (4) Å and with Cremer & Pople (1975) puckering parameters: Q = 0.573 (4) Å, θ = 126.0 (3)° and φ = 11.1 (5)°. In *B*, one ring is essentially planar (Pd1B/N1B/C2B/C3B/C8B/O1B) and two rings have half-chair conformations; Pd1B/N1B/C1B/C17B/C16B/N2B with atom C17B displaced from the Pd1B/N1B/C1B/C16B/N2B plane by -0.443 (4) Å and with Cremer & Pople (1975) puckering parameters: Q = 0.618 (5) Å, θ = 115.8 (4)° and φ = 340.2 (5)°; Pd1B/N2B/C9B/C14B/C15B/O2B with atom O2B deviated from the Pd1B/N2B/C9B/C14B/C15B plane by -0.125 (4) Å and with Cremer & Pople (1975) puckering parameters: Q = 0.199 (5) Å, θ = 62.9 (14)° and φ = 24.4 (17)°. The Pd^II^ ions are coordinated in a *cis*-planar fashion by the two phenolic oxygen atoms and two imine nitrogen atoms. The Pd—O distances are in the range 1.979 (3)–2.008 (4) Å with Pd—N distances 1.981 (3)–2.014 (3) Å, which are typical of the square-planar Pd^II^ complexes of Schiff base ligands (Adrian *et al.*, 2008). The bond angles around Pd^II^ ions indicate that the complex has a distorted square-planar geometry as indicated by the angles O—Pd—O in the range 79.66 (11)–80.54 (16)°, O—Pd—N in the range 92.14 (13)–92.95 (11)° and N—Pd—N in the range 94.92 (12)–94.95 (15)°, deviating substantially from that expected for a regular square-planar geometry. The distortion can be attributed to the restricted bite angle of the Schiff base ligand. Other bond lengths and angles observed in the structure are normal (Allen *et al.*, 1987). The dihedral angles between the two phenolate rings [(C3–C8) and (C9–C14)] of the tetradentate Schiff base ligand are 8.3 (2)° in *A* and 18.5 (3)° in *B*.

In the crystal packing (Fig. 2), the neighbouring complex molecules are stacked approximately along the *b* direction by π···π interactions between the Pd1A/N1A/C2A/C3A/C8A/O1A and Pd1B/N1B/C2B/C3B/C8B/O1B rings with the *Cg*···*Cg* distance of 3.5724 (19) Å. The crystal is stabilized by O—H···O hydrogen bonds involving the solvated ethanol molecule (Table 1).

### Experimental

The title complex was synthesized by dissolving the *N*,*N*'-bis-salicylidene-2,2-dimethylpropane-1,3-diamine ligand (0.9313 g, 3 mmol) in dry ethanol (10 ml). Palladium(II) acetate (0.6735 g, 3 mmol) was then added to the resulting solution and refluxed under nitrogen atmosphere for 5 hr. An orange solid was obtained and washed with cold acetonitrile. Yellow single crystals suitable for *X*-ray structure determination were obtained by recrystallization from a mixture of chloroform/hexane (1:1 *v*/*v*) by slow evaporation of the solvent at room temperature over several weeks. Yield: 80%, *M*.p. 608.1–608.6 K.

### Refinement

All H atoms were placed in calculated positions with d(O—H) = 0.82 Å, *U*_iso_=1.2*U*_eq_, d(C—H) = 0.93 Å, *U*_iso_=1.2*U*_eq_(C) for CH and aromatic, 0.96 Å, *U*_iso_ = 1.5*U*_eq_(C) for CH_3_ atoms. A rotating group model was used for the methyl groups. A large peak on the difference electron density map indicated that the oxygen atom (O5) in the ethanol molecule was disordered. The occupancies of the two disorder components were refined to full convergence yielding a ratio of the major-to-minor components of 0.77 (2):0.23 (2).The highest residual electron density peak is located at 1.17 Å from H19D and the deepest hole is located at 0.76 Å from Pd1A.

### Crystal data

**Table:**

[Pd(C_19_H_20_N_2_O_2_)]·0.5C_2_H_6_O	*F*_000_ = 1784
*M**_r_* = 437.83	*D*_x_ = 1.543 Mg m^−^^3^
Monoclinic, *P*2_1_/*c*	Melting point = 608.1–608.6 K
Hall symbol: -P 2ybc	Mo *K*α radiation λ = 0.71073 Å
*a* = 12.2453 (3) Å	Cell parameters from 11002 reflections
*b* = 13.7334 (3) Å	θ = 1.7–30.0º
*c* = 22.8442 (5) Å	µ = 1.00 mm^−^^1^
β = 101.092 (1)º	*T* = 296 (2) K
*V* = 3769.94 (15) Å^3^	Block, yellow
*Z* = 8	0.38 × 0.33 × 0.23 mm

### Data collection

**Table:**

Bruker SMART APEXII CCD area-detector diffractometer	11002 independent reflections
Radiation source: fine-focus sealed tube	8985 reflections with *I* > 2σ(*I*)
Monochromator: graphite	*R*_int_ = 0.039
Detector resolution: 8.33 pixels mm^-1^	θ_max_ = 30.0º
*T* = 296(2) K	θ_min_ = 1.7º
ω scans	*h* = −17→17
Absorption correction: multi-scan(SADABS; Bruker, 2005)	*k* = −18→19
*T*_min_ = 0.690, *T*_max_ = 0.804	*l* = −32→30
93296 measured reflections	

### Refinement

**Table:**

Refinement on *F*^2^	Secondary atom site location: difference Fourier map
Least-squares matrix: full	Hydrogen site location: inferred from neighbouring sites
*R*[*F*^2^ > 2σ(*F*^2^)] = 0.042	H-atom parameters constrained
*wR*(*F*^2^) = 0.130	*w* = 1/[σ^2^(*F*_o_^2^) + (0.0432*P*)^2^ + 6.804*P*] where *P* = (*F*_o_^2^ + 2*F*_c_^2^)/3
*S* = 1.13	(Δ/σ)_max_ = 0.001
11002 reflections	Δρ_max_ = 0.81 e Å^−^^3^
474 parameters	Δρ_min_ = −0.66 e Å^−^^3^
Primary atom site location: structure-invariant direct methods	Extinction correction: none

### Special details

**Table:**

Geometry. All e.s.d.'s (except the e.s.d. in the dihedral angle between two l.s. planes) are estimated using the full covariance matrix. The cell e.s.d.'s are taken into account individually in the estimation of e.s.d.'s in distances, angles and torsion angles; correlations between e.s.d.'s in cell parameters are only used when they are defined by crystal symmetry. An approximate (isotropic) treatment of cell e.s.d.'s is used for estimating e.s.d.'s involving l.s. planes.
Refinement. Refinement of *F*^2^ against ALL reflections. The weighted *R*-factor *wR* and goodness of fit *S* are based on *F*^2^, conventional *R*-factors *R* are based on *F*, with *F* set to zero for negative *F*^2^. The threshold expression of *F*^2^ > σ(*F*^2^) is used only for calculating *R*-factors(gt) *etc*. and is not relevant to the choice of reflections for refinement. *R*-factors based on *F*^2^ are statistically about twice as large as those based on *F*, and *R*- factors based on ALL data will be even larger.

### Fractional atomic coordinates and isotropic or equivalent isotropic displacement parameters (Å^2^)

**Table:**

	*x*	*y*	*z*	*U*_iso_*/*U*_eq_	Occ. (<1)
Pd1A	0.20617 (2)	0.67812 (2)	0.247364 (11)	0.03888 (8)	
O1A	0.1533 (2)	0.6938 (2)	0.16026 (12)	0.0555 (7)	
O2A	0.0603 (2)	0.6083 (2)	0.23691 (12)	0.0530 (7)	
N1A	0.3445 (2)	0.7573 (2)	0.24716 (13)	0.0404 (6)	
N2A	0.2422 (3)	0.6491 (2)	0.33484 (13)	0.0419 (6)	
C1A	0.4329 (3)	0.7719 (3)	0.30025 (17)	0.0516 (9)	
H1A	0.4694	0.8335	0.2960	0.062*	
H1B	0.4879	0.7209	0.3011	0.062*	
C2A	0.3643 (3)	0.7980 (3)	0.19958 (16)	0.0441 (7)	
H2A	0.4285	0.8357	0.2047	0.053*	
C3A	0.3023 (3)	0.7937 (3)	0.14014 (16)	0.0447 (8)	
C4A	0.3479 (4)	0.8453 (4)	0.09669 (19)	0.0603 (11)	
H4A	0.4132	0.8808	0.1084	0.072*	
C5A	0.2975 (5)	0.8435 (4)	0.0380 (2)	0.0778 (16)	
H5A	0.3267	0.8788	0.0099	0.093*	
C6A	0.2013 (5)	0.7877 (4)	0.0209 (2)	0.0783 (16)	
H6A	0.1680	0.7845	−0.0193	0.094*	
C7A	0.1551 (4)	0.7376 (4)	0.06169 (18)	0.0626 (11)	
H7A	0.0909	0.7011	0.0489	0.075*	
C8A	0.2034 (3)	0.7406 (3)	0.12297 (16)	0.0459 (8)	
C9A	0.0151 (3)	0.5704 (3)	0.27855 (18)	0.0465 (8)	
C10A	−0.0911 (4)	0.5261 (3)	0.2617 (2)	0.0581 (10)	
H10A	−0.1253	0.5246	0.2216	0.070*	
C11A	−0.1441 (4)	0.4854 (3)	0.3035 (2)	0.0651 (12)	
H11A	−0.2133	0.4563	0.2913	0.078*	
C12A	−0.0965 (4)	0.4870 (4)	0.3633 (2)	0.0670 (12)	
H12A	−0.1337	0.4595	0.3911	0.080*	
C13A	0.0048 (4)	0.5287 (3)	0.3815 (2)	0.0574 (10)	
H13A	0.0362	0.5304	0.4219	0.069*	
C14A	0.0640 (3)	0.5701 (3)	0.33940 (17)	0.0455 (8)	
C15A	0.1737 (3)	0.6056 (3)	0.36258 (16)	0.0446 (8)	
H15A	0.1991	0.5959	0.4032	0.054*	
C16A	0.3527 (3)	0.6716 (3)	0.37029 (17)	0.0500 (9)	
H16A	0.4054	0.6238	0.3612	0.060*	
H16B	0.3497	0.6652	0.4122	0.060*	
C17A	0.3951 (3)	0.7722 (3)	0.35969 (16)	0.0449 (8)	
C18A	0.4980 (4)	0.7901 (4)	0.4079 (2)	0.0659 (12)	
H18A	0.4770	0.7919	0.4463	0.099*	
H18B	0.5313	0.8511	0.4006	0.099*	
H18C	0.5506	0.7384	0.4072	0.099*	
C19A	0.3091 (4)	0.8516 (3)	0.3627 (2)	0.0656 (12)	
H19A	0.2863	0.8487	0.4006	0.098*	
H19B	0.2456	0.8419	0.3313	0.098*	
H19C	0.3413	0.9142	0.3581	0.098*	
C20A	0.1426 (9)	0.0756 (7)	0.4083 (5)	0.130 (3)	
H20A	0.1760	0.1273	0.3888	0.156*	0.77 (2)
H20B	0.1380	0.0175	0.3838	0.156*	0.77 (2)
H20C	0.0912	0.0220	0.4033	0.156*	0.23 (2)
H20D	0.1936	0.0613	0.3824	0.156*	0.23 (2)
C21A	0.2108 (10)	0.0561 (9)	0.4695 (4)	0.170 (5)	
H21A	0.2848	0.0373	0.4659	0.256*	
H21B	0.1770	0.0045	0.4881	0.256*	
H21C	0.2141	0.1140	0.4933	0.256*	
O5A	0.0483 (10)	0.1001 (10)	0.4158 (6)	0.166 (6)	0.77 (2)
H5AB	0.0085	0.1116	0.3834	0.249*	0.77 (2)
O5B	0.0875 (19)	0.143 (2)	0.3783 (14)	0.104 (11)	0.23 (2)
H5BA	0.0642	0.1245	0.3441	0.157*	0.23 (2)
Pd1B	0.31413 (3)	0.43936 (2)	0.261629 (14)	0.04845 (9)	
O1B	0.4597 (2)	0.5063 (2)	0.26439 (15)	0.0637 (8)	
O2B	0.3781 (3)	0.4244 (3)	0.34754 (15)	0.0747 (10)	
N1B	0.2659 (3)	0.4668 (2)	0.17540 (15)	0.0490 (7)	
N2B	0.1765 (3)	0.3646 (3)	0.26900 (18)	0.0598 (9)	
C1B	0.1535 (4)	0.4404 (3)	0.1444 (2)	0.0632 (12)	
H1C	0.1489	0.4478	0.1018	0.076*	
H1D	0.1003	0.4850	0.1564	0.076*	
C2B	0.3247 (3)	0.5142 (3)	0.1439 (2)	0.0537 (9)	
H2B	0.2924	0.5225	0.1039	0.064*	
C3B	0.4330 (3)	0.5560 (3)	0.1622 (2)	0.0556 (10)	
C4B	0.4766 (5)	0.6069 (4)	0.1186 (3)	0.0770 (14)	
H4B	0.4361	0.6100	0.0798	0.092*	
C5B	0.5785 (6)	0.6522 (4)	0.1326 (4)	0.098 (2)	
H5B	0.6068	0.6868	0.1038	0.118*	
C6B	0.6377 (5)	0.6453 (4)	0.1902 (4)	0.096 (2)	
H6B	0.7073	0.6746	0.1996	0.115*	
C7B	0.5982 (4)	0.5973 (4)	0.2341 (3)	0.0790 (16)	
H7B	0.6402	0.5949	0.2726	0.095*	
C8B	0.4930 (3)	0.5509 (3)	0.2207 (2)	0.0587 (11)	
C9B	0.3223 (6)	0.4019 (4)	0.3892 (2)	0.0790 (17)	
C10B	0.3738 (7)	0.4180 (5)	0.4496 (3)	0.104 (2)	
H10B	0.4454	0.4434	0.4587	0.125*	
C11B	0.3171 (11)	0.3960 (7)	0.4949 (4)	0.145 (5)	
H11B	0.3519	0.4073	0.5342	0.174*	
C12B	0.2135 (11)	0.3588 (8)	0.4841 (5)	0.150 (5)	
H12B	0.1769	0.3462	0.5154	0.180*	
C13B	0.1631 (8)	0.3400 (5)	0.4271 (3)	0.116 (3)	
H13B	0.0925	0.3123	0.4198	0.139*	
C14B	0.2151 (6)	0.3613 (4)	0.3784 (3)	0.0831 (18)	
C15B	0.1529 (5)	0.3416 (3)	0.3197 (3)	0.0738 (15)	
H15B	0.0866	0.3076	0.3182	0.089*	
C16B	0.0904 (4)	0.3331 (4)	0.2181 (3)	0.0781 (15)	
H16C	0.0253	0.3739	0.2169	0.094*	
H16D	0.0690	0.2670	0.2257	0.094*	
C17B	0.1214 (4)	0.3358 (3)	0.1574 (2)	0.0567 (10)	
C18B	0.0165 (4)	0.3119 (4)	0.1106 (3)	0.0839 (17)	
H18D	0.0330	0.3177	0.0713	0.126*	
H18E	−0.0419	0.3566	0.1147	0.126*	
H18F	−0.0070	0.2466	0.1166	0.126*	
C19B	0.2109 (4)	0.2634 (3)	0.1517 (2)	0.0701 (13)	
H19D	0.2742	0.2737	0.1831	0.105*	
H19E	0.2326	0.2716	0.1138	0.105*	
H19F	0.1831	0.1986	0.1545	0.105*	

### Atomic displacement parameters (Å^2^)

**Table:**

	*U*^11^	*U*^22^	*U*^33^	*U*^12^	*U*^13^	*U*^23^
Pd1A	0.04093 (14)	0.04101 (14)	0.03494 (13)	−0.00268 (10)	0.00789 (10)	0.00081 (10)
O1A	0.0576 (16)	0.0694 (18)	0.0376 (13)	−0.0165 (14)	0.0042 (12)	0.0055 (12)
O2A	0.0497 (15)	0.0664 (18)	0.0427 (14)	−0.0157 (13)	0.0082 (11)	0.0014 (12)
N1A	0.0385 (14)	0.0450 (15)	0.0377 (14)	−0.0013 (12)	0.0070 (11)	0.0010 (12)
N2A	0.0483 (16)	0.0395 (14)	0.0373 (14)	0.0001 (12)	0.0069 (12)	−0.0009 (11)
C1A	0.0415 (19)	0.065 (2)	0.046 (2)	−0.0075 (17)	0.0035 (15)	0.0051 (18)
C2A	0.0438 (18)	0.0477 (19)	0.0418 (17)	−0.0027 (15)	0.0106 (14)	−0.0004 (15)
C3A	0.051 (2)	0.0472 (19)	0.0382 (17)	−0.0010 (16)	0.0137 (15)	0.0007 (14)
C4A	0.067 (3)	0.071 (3)	0.046 (2)	−0.015 (2)	0.0178 (19)	0.0035 (19)
C5A	0.095 (4)	0.097 (4)	0.045 (2)	−0.034 (3)	0.021 (2)	0.009 (2)
C6A	0.100 (4)	0.098 (4)	0.035 (2)	−0.024 (3)	0.008 (2)	0.002 (2)
C7A	0.072 (3)	0.073 (3)	0.041 (2)	−0.019 (2)	0.0058 (19)	0.0010 (19)
C8A	0.051 (2)	0.049 (2)	0.0375 (17)	0.0014 (16)	0.0090 (15)	0.0015 (15)
C9A	0.047 (2)	0.0385 (17)	0.056 (2)	−0.0025 (15)	0.0150 (16)	0.0030 (15)
C10A	0.051 (2)	0.056 (2)	0.068 (3)	−0.0066 (18)	0.016 (2)	0.001 (2)
C11A	0.054 (2)	0.058 (3)	0.086 (3)	−0.010 (2)	0.020 (2)	0.009 (2)
C12A	0.069 (3)	0.061 (3)	0.079 (3)	−0.010 (2)	0.035 (3)	0.012 (2)
C13A	0.067 (3)	0.052 (2)	0.057 (2)	−0.005 (2)	0.024 (2)	0.0073 (18)
C14A	0.051 (2)	0.0357 (17)	0.053 (2)	−0.0005 (15)	0.0186 (16)	0.0023 (15)
C15A	0.060 (2)	0.0359 (17)	0.0392 (17)	0.0020 (15)	0.0132 (16)	0.0006 (13)
C16A	0.052 (2)	0.054 (2)	0.0409 (18)	−0.0038 (17)	0.0004 (15)	0.0084 (16)
C17A	0.0469 (19)	0.0467 (19)	0.0393 (17)	−0.0044 (15)	0.0038 (14)	−0.0029 (14)
C18A	0.062 (3)	0.081 (3)	0.049 (2)	−0.018 (2)	−0.0036 (19)	−0.001 (2)
C19A	0.069 (3)	0.053 (2)	0.076 (3)	0.004 (2)	0.014 (2)	−0.017 (2)
C20A	0.149 (9)	0.095 (6)	0.161 (9)	0.019 (6)	0.066 (7)	−0.002 (6)
C21A	0.214 (12)	0.200 (12)	0.090 (6)	0.054 (10)	0.012 (7)	0.001 (6)
O5A	0.127 (8)	0.172 (10)	0.199 (12)	−0.001 (7)	0.032 (8)	−0.037 (9)
O5B	0.068 (13)	0.12 (2)	0.113 (19)	−0.026 (12)	−0.005 (11)	0.008 (14)
Pd1B	0.04901 (17)	0.04124 (15)	0.05369 (17)	0.00567 (12)	0.00636 (12)	−0.00465 (12)
O1B	0.0474 (16)	0.0648 (19)	0.073 (2)	−0.0018 (14)	−0.0043 (14)	−0.0102 (16)
O2B	0.084 (2)	0.079 (2)	0.0561 (18)	0.0227 (19)	0.0020 (17)	−0.0023 (16)
N1B	0.0443 (17)	0.0384 (15)	0.0600 (19)	−0.0051 (13)	−0.0005 (14)	−0.0031 (14)
N2B	0.067 (2)	0.0447 (18)	0.073 (2)	0.0027 (16)	0.0266 (19)	0.0007 (17)
C1B	0.051 (2)	0.055 (2)	0.076 (3)	−0.0099 (19)	−0.008 (2)	0.006 (2)
C2B	0.052 (2)	0.0412 (19)	0.064 (2)	−0.0044 (16)	0.0034 (18)	−0.0022 (17)
C3B	0.048 (2)	0.0376 (18)	0.083 (3)	−0.0029 (16)	0.016 (2)	−0.0081 (19)
C4B	0.079 (3)	0.055 (3)	0.103 (4)	−0.010 (2)	0.032 (3)	0.003 (3)
C5B	0.086 (4)	0.066 (3)	0.158 (7)	−0.017 (3)	0.060 (5)	−0.002 (4)
C6B	0.057 (3)	0.072 (3)	0.166 (7)	−0.022 (3)	0.039 (4)	−0.027 (4)
C7B	0.044 (2)	0.069 (3)	0.119 (5)	−0.008 (2)	0.002 (3)	−0.029 (3)
C8B	0.043 (2)	0.043 (2)	0.090 (3)	−0.0023 (16)	0.011 (2)	−0.018 (2)
C9B	0.123 (5)	0.059 (3)	0.055 (3)	0.041 (3)	0.016 (3)	0.006 (2)
C10B	0.157 (7)	0.087 (4)	0.063 (3)	0.057 (4)	0.010 (4)	0.005 (3)
C11B	0.262 (13)	0.114 (7)	0.060 (4)	0.102 (9)	0.032 (7)	0.023 (4)
C12B	0.241 (13)	0.130 (8)	0.098 (7)	0.070 (9)	0.083 (9)	0.047 (6)
C13B	0.173 (8)	0.092 (5)	0.099 (5)	0.041 (5)	0.069 (5)	0.041 (4)
C14B	0.122 (5)	0.059 (3)	0.077 (4)	0.039 (3)	0.043 (4)	0.023 (3)
C15B	0.092 (4)	0.050 (2)	0.091 (4)	0.018 (2)	0.046 (3)	0.014 (2)
C16B	0.062 (3)	0.077 (3)	0.098 (4)	−0.021 (3)	0.021 (3)	−0.014 (3)
C17B	0.047 (2)	0.046 (2)	0.076 (3)	−0.0097 (17)	0.0090 (19)	−0.0062 (19)
C18B	0.059 (3)	0.066 (3)	0.117 (5)	−0.022 (2)	−0.005 (3)	−0.006 (3)
C19B	0.071 (3)	0.053 (2)	0.084 (3)	0.002 (2)	0.009 (3)	−0.017 (2)

### Geometric parameters (Å, °)

**Table:**

Pd1A—O1A	1.982 (3)	C21A—H21A	0.9600
Pd1A—O2A	2.001 (3)	C21A—H21B	0.9600
Pd1A—N2A	2.002 (3)	C21A—H21C	0.9600
Pd1A—N1A	2.014 (3)	O5A—H20C	1.2516
O1A—C8A	1.310 (4)	O5A—H5AB	0.8200
O2A—C9A	1.298 (4)	O5B—H5BA	0.8200
N1A—C2A	1.286 (4)	Pd1B—O2B	1.979 (3)
N1A—C1A	1.475 (5)	Pd1B—N1B	1.981 (3)
N2A—C15A	1.292 (5)	Pd1B—O1B	1.995 (3)
N2A—C16A	1.469 (5)	Pd1B—N2B	2.008 (4)
C1A—C17A	1.516 (5)	O1B—C8B	1.302 (6)
C1A—H1A	0.9700	O2B—C9B	1.312 (7)
C1A—H1B	0.9700	N1B—C2B	1.288 (5)
C2A—C3A	1.423 (5)	N1B—C1B	1.468 (5)
C2A—H2A	0.9300	N2B—C15B	1.287 (6)
C3A—C8A	1.405 (5)	N2B—C16B	1.476 (7)
C3A—C4A	1.418 (5)	C1B—C17B	1.533 (6)
C4A—C5A	1.364 (6)	C1B—H1C	0.9700
C4A—H4A	0.9300	C1B—H1D	0.9700
C5A—C6A	1.397 (7)	C2B—C3B	1.432 (6)
C5A—H5A	0.9300	C2B—H2B	0.9300
C6A—C7A	1.366 (6)	C3B—C8B	1.397 (7)
C6A—H6A	0.9300	C3B—C4B	1.403 (7)
C7A—C8A	1.412 (5)	C4B—C5B	1.376 (8)
C7A—H7A	0.9300	C4B—H4B	0.9300
C9A—C14A	1.404 (6)	C5B—C6B	1.379 (10)
C9A—C10A	1.420 (6)	C5B—H5B	0.9300
C10A—C11A	1.374 (6)	C6B—C7B	1.363 (9)
C10A—H10A	0.9300	C6B—H6B	0.9300
C11A—C12A	1.379 (7)	C7B—C8B	1.417 (6)
C11A—H11A	0.9300	C7B—H7B	0.9300
C12A—C13A	1.357 (7)	C9B—C14B	1.404 (9)
C12A—H12A	0.9300	C9B—C10B	1.418 (8)
C13A—C14A	1.429 (5)	C10B—C11B	1.387 (12)
C13A—H13A	0.9300	C10B—H10B	0.9300
C14A—C15A	1.431 (5)	C11B—C12B	1.346 (15)
C15A—H15A	0.9300	C11B—H11B	0.9300
C16A—C17A	1.513 (5)	C12B—C13B	1.354 (13)
C16A—H16A	0.9700	C12B—H12B	0.9300
C16A—H16B	0.9700	C13B—C14B	1.415 (8)
C17A—C18A	1.526 (5)	C13B—H13B	0.9300
C17A—C19A	1.526 (6)	C14B—C15B	1.434 (9)
C18A—H18A	0.9600	C15B—H15B	0.9300
C18A—H18B	0.9600	C16B—C17B	1.507 (7)
C18A—H18C	0.9600	C16B—H16C	0.9700
C19A—H19A	0.9600	C16B—H16D	0.9700
C19A—H19B	0.9600	C17B—C19B	1.504 (6)
C19A—H19C	0.9600	C17B—C18B	1.541 (6)
C20A—O5A	1.246 (13)	C18B—H18D	0.9600
C20A—O5B	1.27 (3)	C18B—H18E	0.9600
C20A—C21A	1.508 (13)	C18B—H18F	0.9600
C20A—H20A	0.9700	C19B—H19D	0.9600
C20A—H20B	0.9700	C19B—H19E	0.9600
C20A—H20C	0.9598	C19B—H19F	0.9600
C20A—H20D	0.9599		
			
O1A—Pd1A—O2A	79.66 (11)	O5B—C20A—H20D	99.0
O1A—Pd1A—N2A	172.05 (12)	C21A—C20A—H20D	103.0
O2A—Pd1A—N2A	92.53 (12)	H20A—C20A—H20D	58.9
O1A—Pd1A—N1A	92.95 (11)	H20B—C20A—H20D	56.5
O2A—Pd1A—N1A	172.15 (11)	H20C—C20A—H20D	105.0
N2A—Pd1A—N1A	94.92 (12)	C20A—C21A—H21A	109.5
C8A—O1A—Pd1A	127.1 (2)	C20A—C21A—H21B	109.5
C9A—O2A—Pd1A	127.0 (2)	H21A—C21A—H21B	109.5
C2A—N1A—C1A	114.1 (3)	C20A—C21A—H21C	109.5
C2A—N1A—Pd1A	122.1 (3)	H21A—C21A—H21C	109.5
C1A—N1A—Pd1A	123.7 (2)	H21B—C21A—H21C	109.5
C15A—N2A—C16A	116.3 (3)	C20A—O5A—H20C	45.2
C15A—N2A—Pd1A	122.9 (3)	C20A—O5A—H5AB	109.5
C16A—N2A—Pd1A	120.8 (2)	H20C—O5A—H5AB	99.3
N1A—C1A—C17A	115.8 (3)	C20A—O5B—H5BA	109.5
N1A—C1A—H1A	108.3	O2B—Pd1B—N1B	172.48 (15)
C17A—C1A—H1A	108.3	O2B—Pd1B—O1B	80.54 (16)
N1A—C1A—H1B	108.3	N1B—Pd1B—O1B	92.14 (13)
C17A—C1A—H1B	108.3	O2B—Pd1B—N2B	92.43 (17)
H1A—C1A—H1B	107.4	N1B—Pd1B—N2B	94.95 (15)
N1A—C2A—C3A	129.5 (4)	O1B—Pd1B—N2B	172.66 (15)
N1A—C2A—H2A	115.2	C8B—O1B—Pd1B	127.0 (3)
C3A—C2A—H2A	115.2	C9B—O2B—Pd1B	125.6 (4)
C8A—C3A—C4A	119.9 (4)	C2B—N1B—C1B	115.4 (4)
C8A—C3A—C2A	124.1 (3)	C2B—N1B—Pd1B	124.1 (3)
C4A—C3A—C2A	116.0 (4)	C1B—N1B—Pd1B	120.4 (3)
C5A—C4A—C3A	121.0 (4)	C15B—N2B—C16B	112.7 (5)
C5A—C4A—H4A	119.5	C15B—N2B—Pd1B	122.6 (4)
C3A—C4A—H4A	119.5	C16B—N2B—Pd1B	124.6 (3)
C4A—C5A—C6A	118.9 (4)	N1B—C1B—C17B	112.9 (4)
C4A—C5A—H5A	120.6	N1B—C1B—H1C	109.0
C6A—C5A—H5A	120.6	C17B—C1B—H1C	109.0
C7A—C6A—C5A	121.6 (4)	N1B—C1B—H1D	109.0
C7A—C6A—H6A	119.2	C17B—C1B—H1D	109.0
C5A—C6A—H6A	119.2	H1C—C1B—H1D	107.8
C6A—C7A—C8A	120.8 (4)	N1B—C2B—C3B	128.7 (4)
C6A—C7A—H7A	119.6	N1B—C2B—H2B	115.6
C8A—C7A—H7A	119.6	C3B—C2B—H2B	115.6
O1A—C8A—C3A	124.1 (3)	C8B—C3B—C4B	120.1 (4)
O1A—C8A—C7A	118.1 (4)	C8B—C3B—C2B	123.1 (4)
C3A—C8A—C7A	117.8 (3)	C4B—C3B—C2B	116.7 (5)
O2A—C9A—C14A	124.4 (3)	C5B—C4B—C3B	120.8 (6)
O2A—C9A—C10A	118.0 (4)	C5B—C4B—H4B	119.6
C14A—C9A—C10A	117.5 (4)	C3B—C4B—H4B	119.6
C11A—C10A—C9A	121.1 (4)	C4B—C5B—C6B	118.5 (6)
C11A—C10A—H10A	119.5	C4B—C5B—H5B	120.8
C9A—C10A—H10A	119.5	C6B—C5B—H5B	120.8
C10A—C11A—C12A	121.1 (4)	C7B—C6B—C5B	122.7 (5)
C10A—C11A—H11A	119.4	C7B—C6B—H6B	118.7
C12A—C11A—H11A	119.4	C5B—C6B—H6B	118.7
C13A—C12A—C11A	119.8 (4)	C6B—C7B—C8B	119.7 (6)
C13A—C12A—H12A	120.1	C6B—C7B—H7B	120.2
C11A—C12A—H12A	120.1	C8B—C7B—H7B	120.2
C12A—C13A—C14A	121.0 (4)	O1B—C8B—C3B	124.9 (4)
C12A—C13A—H13A	119.5	O1B—C8B—C7B	116.9 (5)
C14A—C13A—H13A	119.5	C3B—C8B—C7B	118.2 (5)
C9A—C14A—C13A	119.5 (4)	O2B—C9B—C14B	124.4 (5)
C9A—C14A—C15A	123.8 (3)	O2B—C9B—C10B	118.2 (7)
C13A—C14A—C15A	116.6 (4)	C14B—C9B—C10B	117.4 (6)
N2A—C15A—C14A	128.9 (3)	C11B—C10B—C9B	119.9 (9)
N2A—C15A—H15A	115.5	C11B—C10B—H10B	120.0
C14A—C15A—H15A	115.5	C9B—C10B—H10B	120.0
N2A—C16A—C17A	114.3 (3)	C12B—C11B—C10B	122.4 (10)
N2A—C16A—H16A	108.7	C12B—C11B—H11B	118.8
C17A—C16A—H16A	108.7	C10B—C11B—H11B	118.8
N2A—C16A—H16B	108.7	C11B—C12B—C13B	119.2 (9)
C17A—C16A—H16B	108.7	C11B—C12B—H12B	120.4
H16A—C16A—H16B	107.6	C13B—C12B—H12B	120.4
C16A—C17A—C1A	108.3 (3)	C12B—C13B—C14B	121.8 (10)
C16A—C17A—C18A	107.0 (3)	C12B—C13B—H13B	119.1
C1A—C17A—C18A	107.1 (3)	C14B—C13B—H13B	119.1
C16A—C17A—C19A	112.6 (4)	C9B—C14B—C13B	119.3 (7)
C1A—C17A—C19A	112.0 (4)	C9B—C14B—C15B	123.4 (5)
C18A—C17A—C19A	109.5 (4)	C13B—C14B—C15B	117.2 (7)
C17A—C18A—H18A	109.5	N2B—C15B—C14B	128.6 (6)
C17A—C18A—H18B	109.5	N2B—C15B—H15B	115.7
H18A—C18A—H18B	109.5	C14B—C15B—H15B	115.7
C17A—C18A—H18C	109.5	N2B—C16B—C17B	116.6 (4)
H18A—C18A—H18C	109.5	N2B—C16B—H16C	108.1
H18B—C18A—H18C	109.5	C17B—C16B—H16C	108.1
C17A—C19A—H19A	109.5	N2B—C16B—H16D	108.1
C17A—C19A—H19B	109.5	C17B—C16B—H16D	108.1
H19A—C19A—H19B	109.5	H16C—C16B—H16D	107.3
C17A—C19A—H19C	109.5	C19B—C17B—C16B	112.4 (4)
H19A—C19A—H19C	109.5	C19B—C17B—C1B	112.6 (4)
H19B—C19A—H19C	109.5	C16B—C17B—C1B	108.7 (4)
O5A—C20A—O5B	57.8 (12)	C19B—C17B—C18B	108.8 (4)
O5A—C20A—C21A	106.5 (11)	C16B—C17B—C18B	108.0 (4)
O5B—C20A—C21A	140.6 (15)	C1B—C17B—C18B	106.0 (4)
O5A—C20A—H20A	110.4	C17B—C18B—H18D	109.5
O5B—C20A—H20A	56.1	C17B—C18B—H18E	109.5
C21A—C20A—H20A	110.4	H18D—C18B—H18E	109.5
O5A—C20A—H20B	110.4	C17B—C18B—H18F	109.5
O5B—C20A—H20B	109.0	H18D—C18B—H18F	109.5
C21A—C20A—H20B	110.4	H18E—C18B—H18F	109.5
H20A—C20A—H20B	108.6	C17B—C19B—H19D	109.5
O5A—C20A—H20C	67.7	C17B—C19B—H19E	109.5
O5B—C20A—H20C	103.3	H19D—C19B—H19E	109.5
C21A—C20A—H20C	102.0	C17B—C19B—H19F	109.5
H20A—C20A—H20C	146.1	H19D—C19B—H19F	109.5
H20B—C20A—H20C	48.5	H19E—C19B—H19F	109.5
O5A—C20A—H20D	150.5		
			
O2A—Pd1A—O1A—C8A	178.3 (4)	O2B—Pd1B—O1B—C8B	−176.0 (4)
N1A—Pd1A—O1A—C8A	1.0 (3)	N1B—Pd1B—O1B—C8B	2.3 (3)
N2A—Pd1A—O2A—C9A	5.3 (3)	O1B—Pd1B—O2B—C9B	163.4 (4)
O1A—Pd1A—O2A—C9A	−176.2 (3)	N2B—Pd1B—O2B—C9B	−18.7 (4)
O1A—Pd1A—N1A—C2A	2.5 (3)	O1B—Pd1B—N1B—C2B	−0.9 (3)
N2A—Pd1A—N1A—C2A	−178.6 (3)	N2B—Pd1B—N1B—C2B	−179.0 (3)
O1A—Pd1A—N1A—C1A	−175.8 (3)	O1B—Pd1B—N1B—C1B	−175.4 (3)
N2A—Pd1A—N1A—C1A	3.1 (3)	N2B—Pd1B—N1B—C1B	6.5 (3)
O2A—Pd1A—N2A—C15A	−4.4 (3)	O2B—Pd1B—N2B—C15B	10.8 (4)
N1A—Pd1A—N2A—C15A	173.1 (3)	N1B—Pd1B—N2B—C15B	−167.8 (4)
O2A—Pd1A—N2A—C16A	171.8 (3)	O2B—Pd1B—N2B—C16B	−171.4 (4)
N1A—Pd1A—N2A—C16A	−10.7 (3)	N1B—Pd1B—N2B—C16B	10.1 (4)
C2A—N1A—C1A—C17A	150.9 (4)	C2B—N1B—C1B—C17B	136.8 (4)
Pd1A—N1A—C1A—C17A	−30.7 (5)	Pd1B—N1B—C1B—C17B	−48.2 (5)
C1A—N1A—C2A—C3A	175.3 (4)	C1B—N1B—C2B—C3B	175.0 (4)
Pd1A—N1A—C2A—C3A	−3.2 (6)	Pd1B—N1B—C2B—C3B	0.3 (6)
N1A—C2A—C3A—C8A	−0.3 (7)	N1B—C2B—C3B—C8B	−0.4 (7)
N1A—C2A—C3A—C4A	−178.5 (4)	N1B—C2B—C3B—C4B	−178.1 (4)
C8A—C3A—C4A—C5A	−0.5 (7)	C8B—C3B—C4B—C5B	0.1 (7)
C2A—C3A—C4A—C5A	177.8 (5)	C2B—C3B—C4B—C5B	177.8 (5)
C3A—C4A—C5A—C6A	−1.7 (9)	C3B—C4B—C5B—C6B	1.0 (9)
C4A—C5A—C6A—C7A	2.1 (10)	C4B—C5B—C6B—C7B	−1.5 (10)
C5A—C6A—C7A—C8A	−0.1 (9)	C5B—C6B—C7B—C8B	0.9 (9)
Pd1A—O1A—C8A—C3A	−4.3 (6)	Pd1B—O1B—C8B—C3B	−3.0 (6)
Pd1A—O1A—C8A—C7A	175.8 (3)	Pd1B—O1B—C8B—C7B	177.1 (3)
C4A—C3A—C8A—O1A	−177.4 (4)	C4B—C3B—C8B—O1B	179.5 (4)
C2A—C3A—C8A—O1A	4.4 (6)	C2B—C3B—C8B—O1B	1.8 (7)
C4A—C3A—C8A—C7A	2.5 (6)	C4B—C3B—C8B—C7B	−0.6 (6)
C2A—C3A—C8A—C7A	−175.7 (4)	C2B—C3B—C8B—C7B	−178.3 (4)
C6A—C7A—C8A—O1A	177.7 (5)	C6B—C7B—C8B—O1B	−179.9 (5)
C6A—C7A—C8A—C3A	−2.2 (7)	C6B—C7B—C8B—C3B	0.2 (7)
Pd1A—O2A—C9A—C14A	−1.8 (6)	Pd1B—O2B—C9B—C14B	16.8 (7)
Pd1A—O2A—C9A—C10A	178.3 (3)	Pd1B—O2B—C9B—C10B	−164.2 (4)
O2A—C9A—C10A—C11A	−179.4 (4)	O2B—C9B—C10B—C11B	179.4 (6)
C14A—C9A—C10A—C11A	0.6 (6)	C14B—C9B—C10B—C11B	−1.5 (8)
C9A—C10A—C11A—C12A	0.6 (7)	C9B—C10B—C11B—C12B	0.3 (13)
C10A—C11A—C12A—C13A	−0.5 (8)	C10B—C11B—C12B—C13B	1.5 (16)
C11A—C12A—C13A—C14A	−0.9 (7)	C11B—C12B—C13B—C14B	−2.1 (14)
O2A—C9A—C14A—C13A	178.1 (4)	O2B—C9B—C14B—C13B	180.0 (5)
C10A—C9A—C14A—C13A	−1.9 (6)	C10B—C9B—C14B—C13B	0.9 (7)
O2A—C9A—C14A—C15A	−4.6 (6)	O2B—C9B—C14B—C15B	−1.4 (8)
C10A—C9A—C14A—C15A	175.3 (4)	C10B—C9B—C14B—C15B	179.6 (5)
C12A—C13A—C14A—C9A	2.1 (6)	C12B—C13B—C14B—C9B	0.8 (10)
C12A—C13A—C14A—C15A	−175.4 (4)	C12B—C13B—C14B—C15B	−177.9 (7)
C16A—N2A—C15A—C14A	−176.2 (4)	C16B—N2B—C15B—C14B	−178.9 (5)
Pd1A—N2A—C15A—C14A	0.1 (5)	Pd1B—N2B—C15B—C14B	−0.8 (7)
C9A—C14A—C15A—N2A	5.6 (6)	C9B—C14B—C15B—N2B	−7.3 (8)
C13A—C14A—C15A—N2A	−177.1 (4)	C13B—C14B—C15B—N2B	171.3 (5)
C15A—N2A—C16A—C17A	−136.7 (3)	C15B—N2B—C16B—C17B	−166.5 (4)
Pd1A—N2A—C16A—C17A	46.8 (4)	Pd1B—N2B—C16B—C17B	15.5 (7)
N2A—C16A—C17A—C1A	−75.5 (4)	N2B—C16B—C17B—C19B	66.4 (6)
N2A—C16A—C17A—C18A	169.3 (4)	N2B—C16B—C17B—C1B	−59.0 (6)
N2A—C16A—C17A—C19A	48.9 (5)	N2B—C16B—C17B—C18B	−173.5 (4)
N1A—C1A—C17A—C16A	65.9 (4)	N1B—C1B—C17B—C19B	−47.4 (6)
N1A—C1A—C17A—C18A	−179.0 (4)	N1B—C1B—C17B—C16B	77.9 (5)
N1A—C1A—C17A—C19A	−58.9 (5)	N1B—C1B—C17B—C18B	−166.3 (4)

### Hydrogen-bond geometry (Å, °)

**Table:**

*D*—H···*A*	*D*—H	H···*A*	*D*···*A*	*D*—H···*A*
O5A—H5AB···O1A^i^	0.82	2.33	3.020 (13)	142

Symmetry codes: (i) −*x*, *y*−1/2, −*z*+1/2.
